# RECONSIDERING PROTECTIVE FACTORS OF SUICIDALITY IN OLDER ADULTHOOD: RESULTS FROM THE CDC NVDRS NATIONAL DATASET

**DOI:** 10.1093/geroni/igz038.3153

**Published:** 2019-11-08

**Authors:** Kyle L Bower, Kerstin G Emerson

**Affiliations:** University of Georgia, Athens, Georgia, United States

## Abstract

Existing literature comprehensively addresses the issue of suicide; however, there is still much to be understood concerning this phenomenon in later life. For instance, suicide rates are particularly high for older agricultural workers, yet there remains considerable ambiguity concerning the factors contributing to this public health crisis. We conducted exploratory analyses with the Centers for Disease Control (CDC) National Violent Death Reporting System (NVDRS) 2003-2017 dataset. We identified a sample of individuals who committed suicide while working in the agricultural sector (N= 2,106). We coded “farmer” as anyone who worked in the agricultural sector at their time of death, or prior. The majority (>90%) of the sample was Caucasian male, and approximately 38% was age 65 and older. We also found that 40% of farmers were married (or in a domestic partnership) at the time of death. Specifically, of those 65 and older, 26% were married at the time of death (second largest majority as 70% were widowed). This finding is particularly interesting as marriage or long-term partnerships have been shown in the literature to be a protective health factor in later life, especially for older men. Our findings regarding marital status suggest that having a significant other may not be the most critical relationship for suicide prevention. Future mental health interventions should explore alternative social connections as a means to identify more effective methods of preventing suicide in this vulnerable population.

